# Highly Pathogenic Avian Flu, Japan

**DOI:** 10.3201/eid1007.040116

**Published:** 2004-07

**Authors:** Kazuo Inoue

**Affiliations:** * University of Tokyo, Tokyo, Japan

**Keywords:** Highly Pathogenic Avian Flu, Japan, chickens, H5N1 virus

## Abstract

Highly Pathogenic Avian Flu, Japan

**To the Editor:** More than 15,000 chickens on an egg farm in Yamaguchi Prefecture (Chugoku area) have died since the end of 2003. A highly pathogenic avian influenza virus, which had not appeared in Japan for 79 years, was detected in the dead chickens. Of the 34,600 chickens on the farm, dozens to hundreds have died daily since December 28. Moreover, the deaths have increased during 2004. The Ministry of Agriculture, Forestry, and Fisheries ascertained that the same H5N1 avian influenza virus had caused the bird flu epidemic that started in 1997 in East Asia, including Hong Kong, Vietnam, and South Korea. The H5N1 type is a virulent pathogen that can also infect humans as demonstrated by the >20 deaths in Hong Kong, Vietnam, and Thailand. After the influenza infection was confirmed, the ministry immediately ordered the hennery to recall all eggs that had been shipped. The hennery was then disinfected, and nonworkers were restricted from entering. Yamaguchi Prefecture also restricted transfer of the chickens and eggs within a 30-km radius of the infected hennery. The hennery was the first facility infected in Japan. Since mid-February, an additional three outbreaks have occurred (one in Ohita Prefecture in Kyushu Island and two in Kyoto Prefecture in the Kansai Area). In a big poultry farm in Kyoto, 40,000 deaths of chickens, caused by H5N1, were confirmed. The H5N1 virus was also detected by polymerase chain reaction in crows found dead near the chickens in Kyoto. All four sites with infected chickens are in western Japan.

Modern stock raising that involves breeding a large number of domestic animals and fowl in high density has become a risk factor for large-scale outbreaks. The globalization of the marketplace and easy mobility of people and goods have facilitated the spread of many pathogens. Avirulent pathogens that mutate easily may acquire stronger infectious and toxic properties as confirmed in the influenza pandemic of 1918 (1).

Several possibilities exist for the appearance of avian influenza virus in Japan. First, migratory birds from epidemic disease areas might be the primary vectors of the virus. Yamaguchi Prefecture is located 200 km southeast of South Cholla Province, South Korea, where avian influenza is epidemic. The two areas are close enough for wild birds to cross the Korean Strait. Ito et al. reported that avirulent viruses found in wild waterfowl and bearing the consensus avirulence type sequence R-E-T-R have the potential to become pathogenic when present in chickens (2). Thus, migratory birds that are asymptomatic carriers may cross the Korean Strait harboring the H5N1-type virulent viruses generated in Korea. Alternatively, people, cars, and feed grains instead of migratory birds could carry the virulent viruses. To identify the source of infection, the genetic sequence of the virus will be compared with the sequences of viruses acquired in other epidemic areas.

The avian influenza virus did not originally infect other animals, including humans. The virus in Japan had different DNA sequencing from the viruses responsible for human deaths in Hong Kong and Vietnam. However, mutations of the virus in pigs as a result of hybridization are possible, since both avian and human influenza viruses can infect pigs. According to the Food and Agriculture Organization of the United Nations, the H5N1-type virus was detected in pigs raised on farms that also raise chickens infected with the virus in Vietnam. Thus, a new virus that can infect other animals may emerge. In fact, a clouded leopard died of avian influenza in Thailand.

The worst scenario would be that the new virus could be spread from person to person. An avian influenza vaccine is not available in Japan. Because a vaccine may not be developed quickly enough, this new influenza might become pandemic. Therefore, to prevent the virus from infecting humans, bird-to-bird transmission must be stopped.
